# Exploring the conformational landscapes of protein kinases: perspectives from FRET and DEER

**DOI:** 10.1042/BST20230558

**Published:** 2024-05-23

**Authors:** Zachary D. Baker, Damien M. Rasmussen, Nicholas M. Levinson

**Affiliations:** 1 Department of Biochemistry, Molecular Biology and Biophysics, University of Minnesota, Minneapolis, MN 55455, U.S.A.; 2 Department of Pharmacology, University of Minnesota, Minneapolis, MN 55455, U.S.A.

**Keywords:** EPR spectroscopy, fluorescence resonance energy transferscence, protein conformation, protein kinases

## Abstract

Conformational changes of catalytically-important structural elements are a key feature of the regulation mechanisms of protein kinases and are important for dictating inhibitor binding modes and affinities. The lack of widely applicable methods for tracking kinase conformational changes in solution has hindered our understanding of kinase regulation and our ability to design conformationally selective inhibitors. Here we provide an overview of two recently developed methods that detect conformational changes of the regulatory activation loop and αC-helix of kinases and that yield complementary information about allosteric mechanisms. An intramolecular Förster resonance energy transfer-based approach provides a scalable platform for detecting and classifying structural changes in high-throughput, as well as quantifying ligand binding cooperativity, shedding light on the energetics governing allostery. The pulsed electron paramagnetic resonance technique double electron-electron resonance provides lower throughput but higher resolution information on structural changes that allows for unambiguous assignment of conformational states and quantification of population shifts. Together, these methods are shedding new light on kinase regulation and drug interactions and providing new routes for the identification of novel kinase inhibitors and allosteric modulators.

## Introduction

Protein phosphorylation on serine, threonine, and tyrosine residues by eukaryotic protein kinases plays a central role in the signaling pathways controlling the growth and proliferation of cells [1]. The fidelity of kinase signal transduction relies upon stringent regulatory mechanisms that restrain kinase activity, and disruption of these regulation mechanisms is a major driver of human cancer [2]. Advances in our understanding of kinase regulatory processes have been instrumental in spurring the development of targeted kinase inhibitors that now represent standard-of-care treatments for many cancers [3].

**Figure 1.**
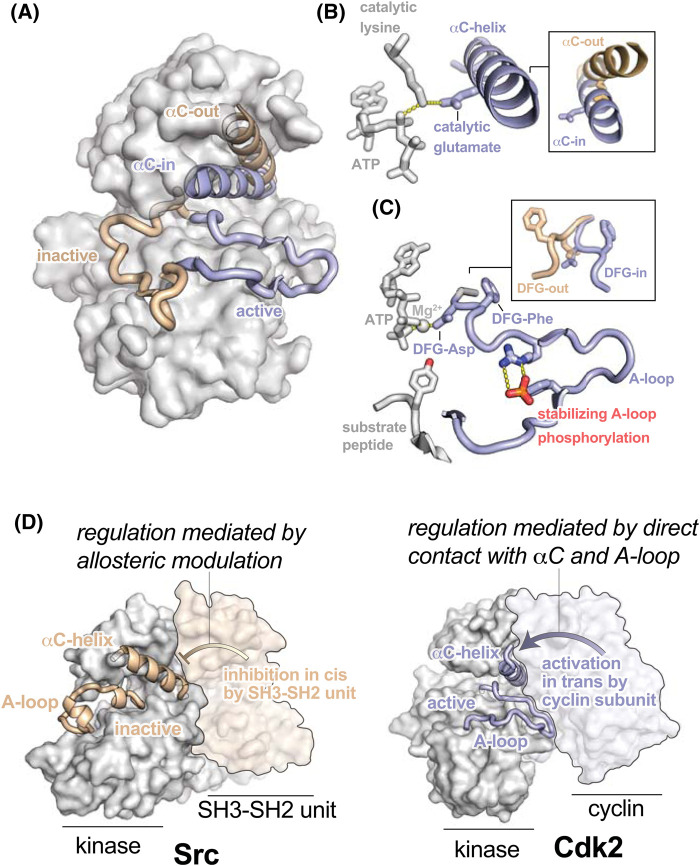
Structural rearrangements accompanying kinase activation. (**A**) Structures of active (blue, PDB ID: 1IR3) and inactive (wheat, PDB ID: 1IRK) insulin receptor kinase are shown, highlighting the different conformations of the A-loop and αC-helix. (**B**) Figure showing the ionic interaction between the glutamate residue on the αC-helix and the catalytic lysine residue in the active site. The inset shows a comparison of the αC-in and αC-out states. (**C**) Figure showing the active conformation of the A-loop (blue) with peptide substrate bound and activating phosphorylation on the A-loop (red). The DFG aspartate residue participates in key ionic interactions that serve to coordinate and position Mg^2+^-ATP. The inset shows the conformational change of the DFG motif between DFG-in and DFG-out conformations. (**D**) Structures of inactive Src (wheat, PDB ID: 2SRC) and active Cdk2 kinases (blue, PDB ID: 1JST) highlighting the role of regulatory interactions in modulating kinase activity by stabilizing the inactive or active states, respectively.

Kinase regulation is typically mediated by conformational rearrangements of two dynamic structural elements within the catalytic domain, the activation loop (A-loop) and the αC-helix (Figure 1A) [4,5]. In activated kinases, these elements play a critical role in promoting catalysis. The N-terminal end of the A-loop contains the catalytic Asp-Phe-Gly (DFG) motif, while the C-terminal end comprises the primary docking site for protein substrates. The kinase αC-helix, in turn, contains a critical glutamate residue that forms a salt bridge with a catalytic lysine (Figure 1B). Correct positioning of both the DFG Asp and catalytic Lys is essential for coordination of Mg^2+^-ATP and transfer of phosphate to the protein substrate (Figure 1B,C) [6,7].

When kinase activity is down-regulated both the A-loop and αC-helix can undergo dramatic rearrangements into inactive conformations. While the A-loop adopts a range of inactive conformations in different kinases, these conformations share the general features that Mg^2+^-ATP and/or protein substrate binding are disrupted (Figure 1A). Additionally, adoption of inactive A-loop conformations is often but not always coupled to a specific rearrangement of the DFG motif from a ‘DFG-in’ conformation, where the DFG-Asp residue points into the active site, to a ‘DFG-out’ conformation where the DFG-Asp and DFG-Phe swap positions [8], thereby preventing Mg^2+^ coordination (Figure 1C, inset). In many kinases the αC-helix can pivot away from the active ‘αC-in’ state, in which the Glu-Lys salt bridge is formed, and adopt an ‘αC-out’ state where the glutamate residue is rotated out of the active site and the salt bridge is broken (Figure 1B).

Many kinase regulation mechanisms work by tuning the populations of active and inactive conformational states of the kinase domain. Two of the most common regulatory events are phosphorylation of regulatory sites on the kinase, and binding of regulatory proteins or second messengers [7]. A large fraction of both serine/threonine and tyrosine kinases are activated by phosphorylation on a conserved site within the A-loop that stabilizes the active conformation (Figure 1C) and is mediated either by autophosphorylation, such as in Src [9], or by another kinase, such as the CDK-activating kinase (CAK) that phosphorylates the cyclin-dependent kinases [10]. Regulatory interactions may impinge directly on the A-loop or αC-helix, locking them in particular conformations, or alternatively on distal surfaces of the kinase, stabilizing specific states allosterically. These regulatory inputs can occur in *cis*, utilizing segments or domains external to the kinase domain, or in *trans* with other protein partners (Figure 1D).

Genetic alterations in kinase genes are a major driver of human cancer [11]. Oncogenic kinase mutations often perturb the conformational balance of the kinase, destabilizing inactive or stabilizing active states. For example, the V600E mutation in BRAF (melanoma) and the L858R mutation in EGFR (non-small cell lung cancer), are thought to destabilize the inactive αC-out conformation, creating a constitutively active kinase [12,13]. Oncogenic fusions of kinase genes can result in similar dysregulation such as in the case of the BCR-Abl fusion in chronic myeloid leukemia (CML), which disrupts autoinhibition through regulatory domain truncations, inducing constitutive kinase activity [14].

To date there are 80+ FDA-approved small-molecule kinase inhibitors [15] that target kinases from 21 different kinase families [16], mostly for the treatment of cancer. Clinical responses to these targeted therapies can be profound, with high rates of response and near-complete initial remissions, as exemplified by the BCR-Abl inhibitor imatinib in CML [17] and the BRAF inhibitor vemurafenib in melanoma [18]. Unfortunately, these responses are usually short lived, with clinical resistance developing within a year in most cases.

Kinase inhibitor resistance is frequently associated with mutations in the kinase domain [19], but can also occur through alterations elsewhere in the protein or via compensatory rewiring of signaling pathways [20,21]. A particularly widespread mechanism in many kinases is mutation of the active site ‘gatekeeper’ residue, for instance T315I in BCR-Abl and T790M in EGFR, which sterically blocks binding of first-generation inhibitors [22,23]. ATP-competitive kinase inhibitors are classified based on the area of the active site which they occupy [24] as well as the conformation of the kinase that they stabilize. Type-I inhibitors tend to occupy a minimal footprint in the active site of the kinase and can prefer either the DFG-in or DFG-out conformations. In contrast, type-II inhibitors occupy the same binding site as type I inhibitors but additionally extend into a hydrophobic backpocket that is unique to the inactive DFG-out state [23], while type 1.5 inhibitors recognize the αC-out conformation [25]. Inhibitor resistance may involve elimination of the binding-competent state [26], or more subtle perturbations to the conformational landscape that allosterically weaken inhibitor binding [27] and prevent effective inhibition [28].

**Figure 2.**
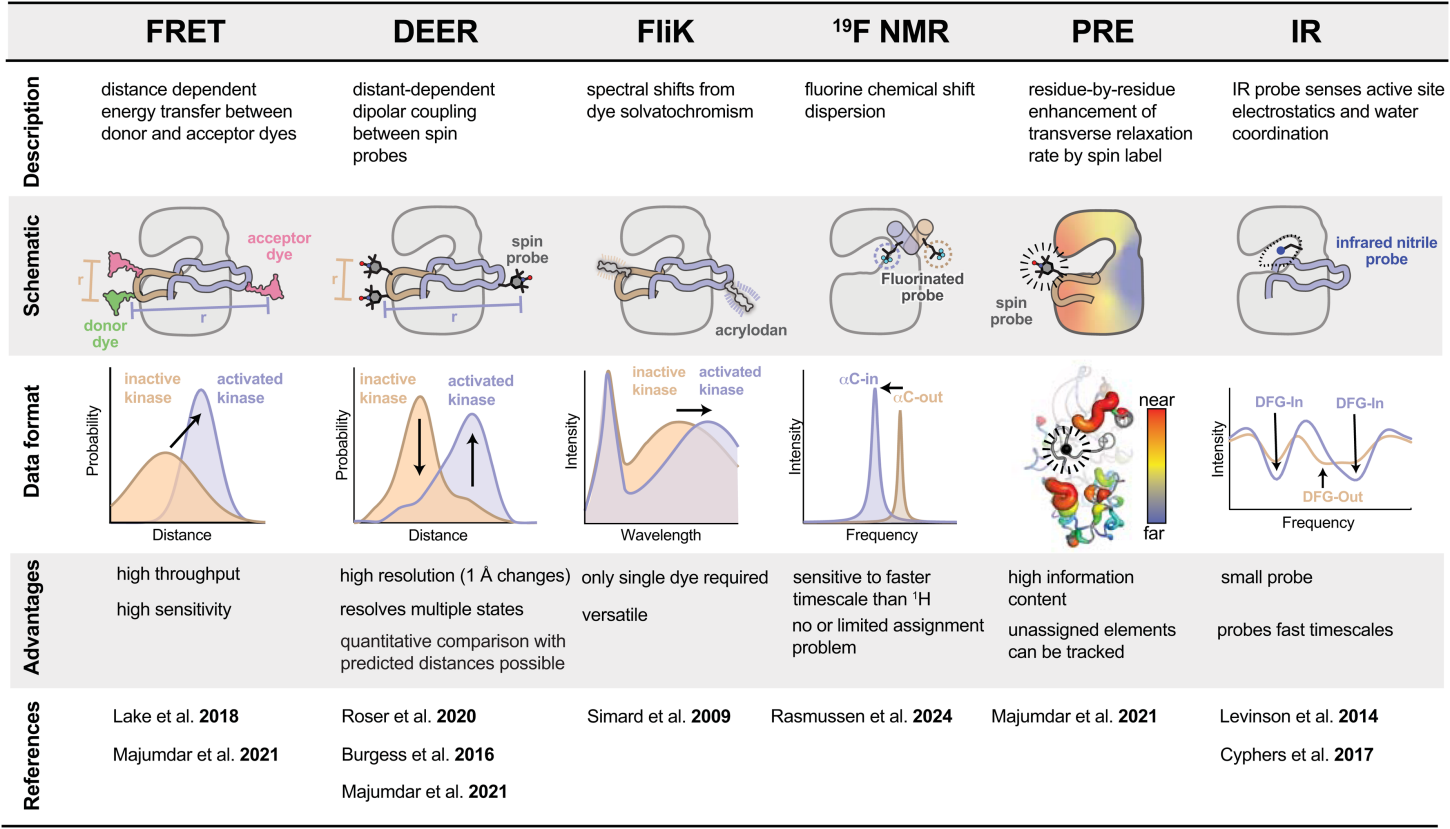
Overview of spectroscopic techniques for probing kinase structural changes. Table showing descriptions, schematic representations, example data format, primary advantages and key references for six different methods utilized to track kinase conformational changes and ligand binding.

It should be evident from the above that the conformational landscapes of protein kinases are of profound importance for kinase biology, drug targeting, and drug resistance mechanisms. While X-ray structures have provided static snapshots of regulatory interactions and allowed specific conformational states to be visualized, they fail to capture the energetics and dynamics of kinase regulation, such as the degree to which conformational states are sampled and how conformational shifts mediate regulation. While solution NMR spectroscopy, the preeminent biophysical technique for probing protein dynamics, has provided important insights in specific cases [29–31], it has proven exceptionally challenging to apply to many kinases because the time scales of the motions of the A-loop and αC-helix fall into the intermediate exchange regime, resulting in severe line broadening of NMR signals [32,33]. Recently, several alternative approaches have been developed by our group and others that have helped catalyze new discoveries regarding the mechanisms of kinase regulation. These approaches are based on an array of spectroscopic techniques including infrared spectroscopy [34], paramagnetic relaxation enhancement [35], electron spin resonance [36–38], fluorine NMR [39], and fluorescence [40–44] (Figure 2). Here, we highlight two complementary and broadly applicable approaches from within this set, double electron-electron resonance (DEER) and Förster resonance energy transfer (FRET), and provide a practical guide for their use. We hope that these approaches will help provide new opportunities for exploring kinase conformational landscapes, will advance our mechanistic understanding of kinase-driven disease and kinase inhibition, and inspire new strategies for the development of next-generation kinase inhibitors.

## Intramolecular FRET-based approaches

**Figure 3.**
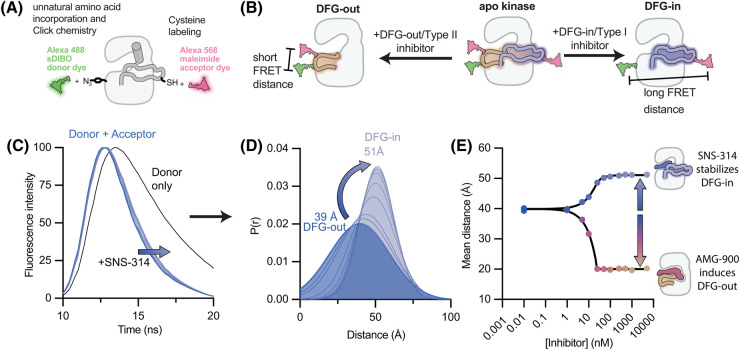
Obtaining inhibitor binding mode assignments by time-resolved FRET. (**A**) Schematic illustrating labeling strategies for incorporating two fluorescent dyes into the kinase. Cysteine labeling can be used either alone or in combination with unnatural amino acid incorporation and Click chemistry for site-specific labeling. (**B**) Schematic representation of the FRET distances expected for different A-loop conformations of the kinase. (**C**) Representative time-resolved fluorescence decays for labeled Aurora kinase A. The black curve represents the donor-only control sample lacking acceptor dye, the blue curves are acceptor + donor labeled sample containing different concentrations of DFG-in/Type I inhibitor SNS-314, with lighter blue indicating higher concentrations. (**D**) FRET distance distributions obtained by global fitting of the time-resolved fluorescence data to a Gaussian distance model. (**E**) The mean distances obtained from fitting are plotted as a function of inhibitor concentration for DFG-in/Type I inhibitor SNS-314 and DFG-out/Type II inhibitor AMG-900. Data were fit to the quadratic solution to the 1:1 binding relationship.

Residues within the kinase A-loop move by up to 30 Å between active and inactive states, a scale of structural change well suited for detection by FRET, and the high sensitivity of fluorescence lends itself to the development of scalable and high-throughput assays. The feasibility of resolving active and inactive A-loop conformations using intramolecular FRET was first demonstrated for the mitotic kinase Aurora A (AurA). These experiments helped to uncover the distinct molecular mechanisms governing activation of AurA by A-loop phosphorylation and by the binding of the mitotic spindle protein TPX2 [34,36]. The intramolecular FRET sensor was constructed by covalently linking a donor (Alexa 488) and acceptor (Alexa 568) dye onto the A-loop itself and a relatively immobile distal site on the kinase domain (Figure 3A). In the following sections we describe how this strategy can be used to track conformational changes and quantify the binding cooperativity of ligands (Figure 3B), and provide guidelines for FRET sensor construction.

### Quantitative conformational measurements by nanosecond time-resolved FRET

A quantitative interpretation of structural rearrangements in the kinase from FRET data is best achieved by employing fluorescence lifetime spectroscopy, where the donor fluorescence decay after pulsed excitation is measured with subnanosecond time resolution [45,46]. Fluorescence lifetime data (Figure 3C) can be fit to a model comprising a continuous Gaussian distribution of FRET distances (Figure 3D), or to two different distance distributions to model population shifts between structural states. Interpretation of the data in terms of FRET distances and correction for donor-only (non-FRET) effects can be performed simultaneously in a single fit analysis and is more robust than with steady-state fluorescence due to the intensity independence of the fluorescence lifetime. This approach was utilized to identify the directionality and magnitude of A-loop rearrangements triggered by inhibitor binding to AurA using a panel of 24 ATP-competitive inhibitors [41,47]. Stabilization of DFG-in or DFG-out states could be inferred from increases or decreases in FRET distance, respectively (Figure 3D,E). Most of the inhibitors in the panel triggered substantial structural rearrangements upon binding, revealing the ubiquitous nature of inhibitor conformational effects. For type I inhibitors, which are often described as not distinguishing between structural states of the kinase, quantitation of the conformational shifts from a two-state fit revealed that stabilization of the DFG-in state ranged from 0.5 to 2 kcal/mol. Interestingly, for several inhibitors that bind to the DFG-in state of Aurora B, the FRET data demonstrated induction of the DFG-out state in AurA, indicating that inhibitors can switch binding modes even when binding closely related kinases. This study highlighted the power of the A-loop FRET technique for quantifying population shifts driven by inhibitor binding and assigning binding modes in a format suitable for high-throughput assays.

### Mapping ligand cooperativity by ratiometric steady-state FRET

**Figure 4.**
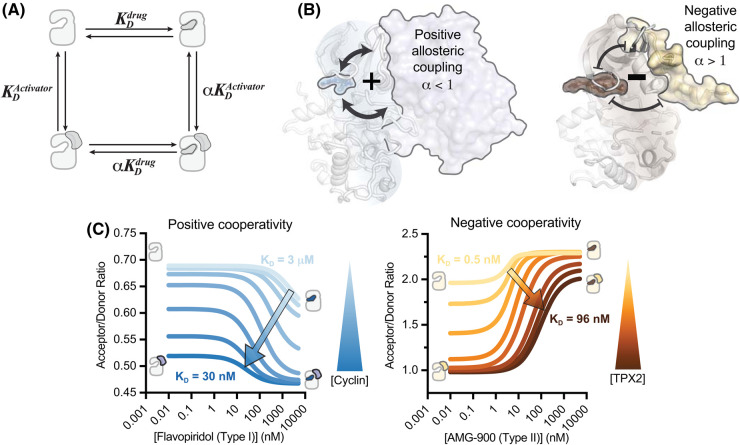
Mapping allosteric coupling by ratiometric steady-state FRET. (**A**) Thermodynamic cycle for two ligands binding to the same kinase, with the parameter α representing the allosteric coupling between the two binding events. (**B**) Visual representation of positive (left, PDB ID: 1JST) and negative (right, 1OL5) allosteric coupling between kinase allosteric modulator proteins and kinase inhibitors. (**C**) ratiometric steady-state FRET results for a type I inhibitor and cyclin binding to Cdk2 (left) and a type II inhibitor and TPX2 binding to Aurora A (right). Each curve represents the best fit curve generated for a single inhibitor titration dataset, with each dataset measured at a different activator protein concentration. The allosteric coupling is readily apparent as a leftwards shift of the binding curves at higher cyclin concentration (left) and a rightwards shift for higher TPX2 concentrations (right). The magnitude of allosteric coupling (α) can be quantified by globally fitting these data to the thermodynamic model shown in panel A.

The same FRET sensor design described above can be paired with steady-state fluorescence measurements as a more accessible alternative to time-resolved detection. Fluorescence emission spectra analyzed in terms of donor-to-acceptor ratio provide a highly robust readout of ligand binding. While ratiometric FRET does not provide direct information on FRET distances, the directionality of observed changes tends to be correlated with the underlying structural change, and the experiments require less sophisticated instrumentation to perform and are more straightforward to analyze. This approach has been used to track ligand-driven structural changes in AurA and the cyclin-dependent kinase Cdk2, allowing the binding cooperativity between inhibitors and kinase binding partners to be quantified (Figure 4A). The results have shed new light on how divergent allosteric regulatory mechanisms differentially affect inhibitor binding (Figure 4B).

In the example of AurA, this approach was used to systematically measure the cooperativity between inhibitor binding and the binding of the AurA activator TPX2 using a global fitting approach to extract cooperativity values (Figure 4C) [41]. A remarkable correspondence was observed between the measured cooperativity and the binding mode assignments obtained by time-resolved FRET, above, with DFG-in/type I inhibitors displaying positive cooperativity with TPX2, which also promotes the DFG-in state, and DFG-out/type II inhibitors exhibiting negative cooperativity with TPX2. In several cases the negative cooperativity was found to be as large as 100-fold (Figure 4C), demonstrating that the energetics of allosteric coupling can be substantial. These large effects on inhibitor affinity would be expected to drive weaker inhibition of the microtubule-associated pool of AurA, which is bound to TPX2, compared with the centrosomally-localized pool, which is not. Similarly large cooperativity effects have also been observed with the cyclin dependent kinase Cdk2 and Cdk inhibitors (Figure 4C) [35] suggesting this is a general feature of kinase/ligand interactions that likely plays a major role in shaping inhibitor recognition across the kinome.

### Considerations for designing FRET sensors to track the kinase A-loop

**Figure 5.**
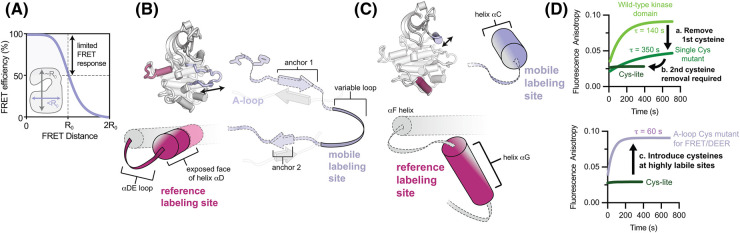
Strategies for probe incorporation for tracking the A-loop and αC-helix. (**A**) The FRET efficiency curve is shown in relation to the scale of a kinase domain in units of the Förster radius *R*_0_, the characteristic distance at which FRET efficiency is 50%. (**B**) For tracking the A-loop the mobile dye can be incorporated onto the tip of the loop to minimize perturbations to the anchoring of the loop onto the C-lobe or to substrate binding. The relatively immobile αD-helix provides a good reference site that optimizes the magnitude of distance changes expected for major conformational changes of the A-loop. (**C**) For tracking the αC-helix the exterior surface of the helix can be labeled but care must be taken to avoid interference with regulatory interactions. Relocating the reference site from the αD to the αG helix may provide superior detection of small conformational changes of the αC-helix. (**D**) Representative stopped flow fluorescence anisotropy measurements illustrating the ability to track the effects of cysteine removal or addition on dye labeling kinetics.

The size of the kinase domain and scale of structural movements impose constraints on the design of intramolecular FRET sensors. Labeling must be performed with small organic dyes, rather than large fluorescent proteins, and because the kinase domain is smaller in diameter than the Förster radius of commomly used FRET dye pairs (50–60 Å), energy transfer efficiencies will be towards the high end of the sensitive range in all structural states, and dyes should be placed as far apart as possible to allow different states to be resolved (Figure 5A). Finally, the critical importance of both the A-loop and αC-helix to kinase function make it imperative that the functional integrity of the FRET sensor be verified experimentally.

A general design strategy for constructing intramolecular FRET sensors to track A-loop movements is to incorporate one dye onto the tip of the A-loop, between the two anchor points that pin the loop to the C-terminal lobe in the active state [48] (Figure 5B). This segment is not conserved in length or sequence across kinases, is usually solvent exposed [49], and moves large distances during transitions between states, maximizing FRET changes while minimizing functional perturbations. To facilitate interpretation of FRET changes, the second dye should ideally be incorporated onto a relatively immobile site distant from the A-loop site and approximately colinear with the primary direction of movement of the mobile A-loop site. In most kinases, the αD-helix, or the αDE-loop that follows it, are ideal locations.

Fluorophore incorporation is most commonly achieved by thiol labeling of cysteine residues using maleimide chemistry, although other methods like unnatural amino acid incorporation [50] can also be employed. To achieve labeling of specific cysteines may necessitate the creation of ‘Cys-lite’ constructs in which endogenous surface-exposed cysteines are removed. Crystal structures are a starting point for targeting cysteines for removal and identifying optimal sites for probe incorporation. In addition, stopped flow fluorescence anisotropy experiments are a complementary tool for comparing the labeling kinetics of endogenous and introduced cysteine residues and determining whether removal of endogenous cysteines is warranted (Figure 5D). Such experiments may reveal that short labeling times can be employed to selectively label particular cysteines without the need to remove endogenous ones [39].

While ratiometric FRET measurements are relatively forgiving of labeling completeness, for quantitative time-resolved FRET measurements dye incorporation must be carefully controlled. In particular, it is desirable that acceptor labeling go to completion to avoid a subpopulation of molecules labeled only with donor, which contribute a long-lifetime component to the lifetime decay that is not modulated by FRET. For charged fluorophores such as the Alexa dyes singly-donor labeled molecules may be purified by ion exchange chromatography prior to labeling with acceptor [34].

## DEER-based approaches

**Figure 6.**
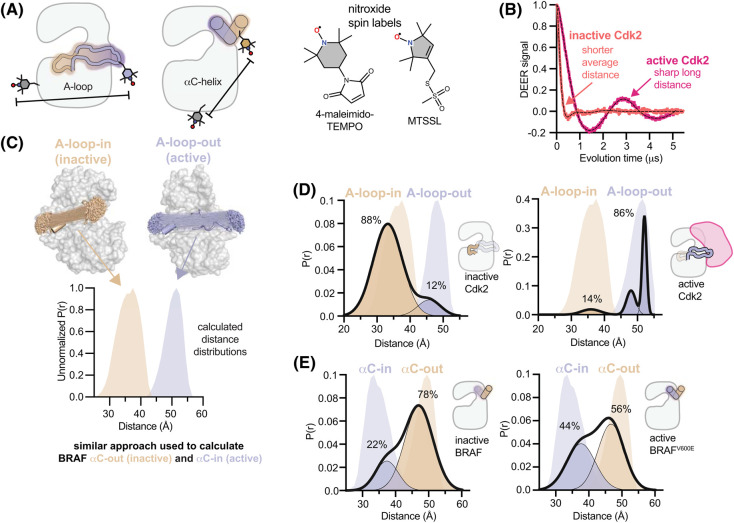
Tracking kinase conformational changes with high resolution by DEER. (**A**) Schematics illustrating kinase labeling with spin labels for tracking the A-loop (left) or αC-helix (right) along with the chemical structures of the commonly used MTSSL and 4-maleimido-TEMPO spin labels. (**B**) Representative dipolar evolution data for Cdk2 in active (magenta) and inactive (salmon) states, highlighting features of the sample apparent in the raw data. (**C**) Representative distance distributions calculated from the X-ray structures of Cdk2 for active (PDB ID: 1JST) and inactive (PDB ID: 1HCK) A-loop conformations using mtsslSuite. A similar analysis was used to generate calculated distance distributions for the αC-in and αC-out states of BRAF. (**D**) Experimental spin-spin distance distributions for inactive unphosphorylated monomeric Cdk2 (left) and for active phosphorylated Cdk2:cyclinA complex (right), obtained by fitting the DEER data shown in panel B. Bold black lines represent the best fit Gaussian model, with the individual populations shown for the inactive (wheat) and active (blue) states. Calculated distance distributions from (**C**) are shown in faded colors behind the experimental data. The conformational shift accompanying kinase activation is observed as a change in the populations of inactive ‘A-loop-in’ and active ‘A-loop-out’ states. (**E**) Experimental spin-spin distance distributions obtained for inactive WT BRAF (left) and BRAF activated by the oncogenic V600E mutation (right) are shown with the same coloring scheme as in panel D.

DEER is a pulsed electron paramagnetic resonance technique that measures the dipolar coupling between unpaired electron spins and provides information about the distribution of spin-spin distances present in the sample [51]. The two spin probes are most commonly nitroxide spin labels with cysteine reactive handles for attachment to the protein of interest, which is then flash frozen and the experiment performed at cryogenic temperatures (Figure 6A). The technique is typically used to measure distances between 15 and 60 Å, and can detect changes as small as 1 Å, making it well suited to studying nuanced conformational changes. Recent studies have used DEER to provide unique perspectives into the conformational ensembles governing the regulation of kinases [35,36,52] and the binding of kinase inhibitors [37,41]. Here we provide two different vignettes that illustrate how this technique can be used to track the kinase A-loop and αC-helix.

### Tracking conformational shifts of the A-loop during CDK2 activation

The activation of the cyclin dependent kinases typically requires both A-loop phosphorylation by CAK and the binding of a cognate cyclin subunit [53]. Unlike cyclin expression, A-loop phosphorylation occurs continuously throughout the cell cycle. However, it cannot activate the kinase by itself and instead assists in activation together with the cyclin subunit, allowing cell cycle progression to be controlled by the timing of cyclin expression, while placing an additional regulatory check on the kinase. DEER experiments on Cdk2 revealed how this is achieved [35]. Using the labeling scheme described below, DEER experiments performed on Cdk2 detected the large-scale (∼20 Å) structural change of the A-loop and allowed the underlying states to be assigned to specific conformations by comparison to distance distributions calculated from the relevant X-ray structures [54,55] (Figure 6B,C). The experiments revealed that in monomeric Cdk2 the conformational ensemble is dominated by the inactive state, with only a 10–15% subpopulation adopting an active-like state, albeit it with a misaligned A-loop that avoids any constitutive kinase activity (Figure 6D). Remarkably, phosphorylation of monomeric Cdk2 on the A-loop did not change the distribution between the two states, indicating that phosphorylation is uncoupled from the activating structural change in the absence of the cyclin subunit. Upon addition of cyclinA however, phosphorylation enhances the conformational shift mediated by the cyclin, thereby locking the kinase in the active state with a correctly aligned A-loop (Figure 6D). This explains why A-loop phosphorylation by CAK is necessary but not sufficient for kinase activation.

### Tracking conformational shifts of the αC-helix in BRAF

The V600E mutation in BRAF confers constitutive kinase activity and is the primary oncogenic driver in several cancers [56]. While X-ray structures suggest that the V600E mutation destabilizes the inactive αC-out state [12], they cannot quantify the degree of destabilization. The sensitivity of DEER allowed the comparatively small (<5 Å) movements of the αC-helix of BRAF to be readily detected and the populations of the αC-in and αC-out states to be quantified (Figure 6E). These experiments showed that apo BRAF favors the inactive αC-out state, with only 22% of the ensemble adopting the active αC-in state. The V600E mutation shifted the conformational equilibrium towards the active state, producing an αC-in population of 44%. This highlights that the V600E mutation causes a surprisingly subtle 2.7-fold shift in the conformational equilibrium towards the active state, which is nonetheless sufficient to confer constitutive activity on normally autoinhibited BRAF monomers. While adoption of the active state of BRAF also promotes kinase dimerization, the conformational shift driven by the V600E mutation is too small to efficiently generate dimers, with the result that the majority of BRAF molecules remain in the monomeric state and are sensitive to monomer-selective RAF inhibitors [57]. Other oncogenic BRAF mutations likely trigger larger conformational shifts that mediate constitutive dimerization, thereby driving resistance to these inhibitors [57].

### Considerations for designing kinase DEER sensors

DEER sensors for tracking A-loop conformation can be designed with a similar architecture as FRET sensors, with spin labels incorporated on the tip of the A-loop and on the αD-helix or αDE-loop (see Figure 5B). For tracking the αC-helix, the mobile labeling site must be moved to the αC-helix itself or the β3-αC loop preceding it. Particular care should be taken to avoid interference with protein:protein interactions that impinge on the αC-helix. Relocating the reference labeling site to the αG-helix may improve the sensitivity to αC-helix motions in some cases (Figure 5C).

As with FRET experiments, the attachment of probes to kinase regulatory regions is constrained by the need to incorporate reactive residues while retaining kinase function. While cysteine labeling is the most common, and there are several commercially available thiol reactive nitroxide spin labels [58], unnatural amino acid incorporation has also been used for site-directed spin labeling [59]. The smaller size of spin probes compared with fluorophores can allow them to reach more buried sites of the kinase, necessitating the removal of cysteine residues that do not react readily with fluorescent dyes.

The optimal protein concentration for DEER experiments is a compromise between achieving strong signal while avoiding intermolecular cross-talk between spins, which in practice restricts samples to the range of 50–100 µM. It is important to establish that sample oligomerization or aggregation do not occur at these concentrations as they can confound the distance measurements. It is also worth noting that deuteration of sample buffer constituents, including glycerol and DMSO if present, substantially enhances the signal and is an important consideration for obtaining high quality data [60].

An advantageous feature of DEER is that direct inspection of the raw background-corrected data prior to fitting reveals important information about the sample, including the average spin-spin distance, which is related to the rate of the signal decay at short evolution times, and whether the sample is rigid with sharply-defined spin-spin distances, which gives rise to readily identifiable signal oscillations that persist at long evolution times (Figure 6B). Fitting of the background-corrected data, using Tikhonov regularization [61] or other techniques [51,62], can provide detailed models of the spin-spin distance distributions responsible for the observed signal. Comparison with theoretical distance distributions predicted from X-ray structures [54,55] often allows reliable assignment of features in the observed distributions to specific structural states (Figure 6C).

### Complementarity of FRET and DEER

While FRET and DEER both provide distance information, the techniques are complementary, with each offsetting weaknesses of the other. For example, the interpretation of DEER data does not suffer from the problem of unknown orientation factor that clouds the interpretation of FRET data [63], rendering DEER superior for determining absolute distances. DEER is also superior for detecting small distance changes, such as those of the αC-helix. Additionally, spin probes tend to be smaller and less perturbing than fluorescent dyes, with fewer rotatable bonds facilitating modeling of probe conformations and predicted distance distributions [54,55]. These advantages come at the cost of far higher sample concentration requirements, lower throughput, and the necessity of studying samples in the frozen state. Performing both FRET and DEER experiments on the same samples can mitigate the limitations of the individual techniques and provide a more in-depth examination of the system.

## Conclusion

The interplay between kinase regulation mechanisms and drug targeting represents an enduring theme in our efforts to elucidate cancer mechanisms and to develop new cancer treatments. It has long been appreciated that conformational changes within the kinase domain play a major role in both kinase regulation and drug recognition, but a quantitative understanding of these phenomena has been hampered by a lack of accessible techniques for tracking these structural changes in solution. The techniques discussed here can address this and are applicable to practically any kinase, while requiring less investment compared with solution NMR spectroscopy, where resonance assignment and exchange broadening of resonances represent major challenges [32].

This approach has catalyzed new insights into regulation mechanisms and inhibitor selectivity in several different kinase systems. One major insight to emerge from these studies is that the allosteric coupling governing kinase regulation spans a broad spectrum of strengths, from subtle conformational shifts triggered by activators or oncogenic mutations [34,36], to wholesale conformational shifts in which the kinase is rigidly locked down in a single state [35]. A second major insight is that conventional ATP-competitive kinase inhibitors have more extensive conformational effects than was previously appreciated [35,41]. Allosteric modulation by kinase inhibitors can be remarkably pleiotropic, with a single inhibitor inducing different conformational states when binding to different kinases, or even when binding to the same kinase complexed with different regulatory partners [41]. The strength of allosteric coupling between regulatory partner proteins and inhibitor binding is sufficient to modulate inhibitor binding affinities by several orders of magnitude. This is similar to the scale of discrimination displayed by most kinase inhibitors across the kinome, indicating that allosteric effects may be as important as sequence differences for shaping inhibitor selectivity patterns. Since allosteric effects are reciprocal [64], inhibitor binding must in turn also modulate the binding of regulatory proteins to a similarly large extent, explaining how inhibitors can trap particular kinase/partner complexes [65,66] or displace specific regulatory partners in a manner that can be critical for efficacy [67,68]. We anticipate that a broader application of techniques for dissecting kinase allostery will continue to uncover new and unexpected roles of allostery in kinase regulation and modulation by inhibitors.

## Perspectives

- The FRET and DEER techniques provide complementary capabilities for mapping the conformational landscapes of protein kinases. Because these techniques detect nearly universal features of kinase regulation they are broadly applicable to practically any protein kinase.
- These techniques are providing new insight into kinase regulation and allosteric modulation by inhibitors. The conformational shifts triggered by oncogenic mutations and inhibitor binding can be quantified, explaining disease mechanisms and defining the allosteric contributions to drug binding and selectivity.
- The high sensitivitiy and scalability of the FRET approach allows it to be translated into a high-throughput screening platform for drug discovery of allosteric modulators. This allows hits with a specific conformational mechanism of action to be directly detected during screening.

## Open Access

Open access for this article was enabled by the participation of University of Minnesota in an all-inclusive *Read & Publish* agreement with Portland Press and the Biochemical Society under a transformative agreement with Individual.

## Abbreviations

CAK: CDK-activating kinase
CML: chronic myeloid leukemia
DEER: double electron-electron resonance
FRET: Förster resonance energy transfer
